# Supplementary material for: Overexpression of Claspin and Timeless protects cancer cells from replication stress in a checkpoint-independent manner
Source: Nat Commun. 2019 Feb 22;10:910. doi: 10.1038/s41467-019-08886-8 (PMC6385232; doi:10.1038/s41467-019-08886-8)
Supplement: Supplementary file 1 — Supplementary Information [file 41467_2019_8886_MOESM1_ESM.pptx]

## Slide 1
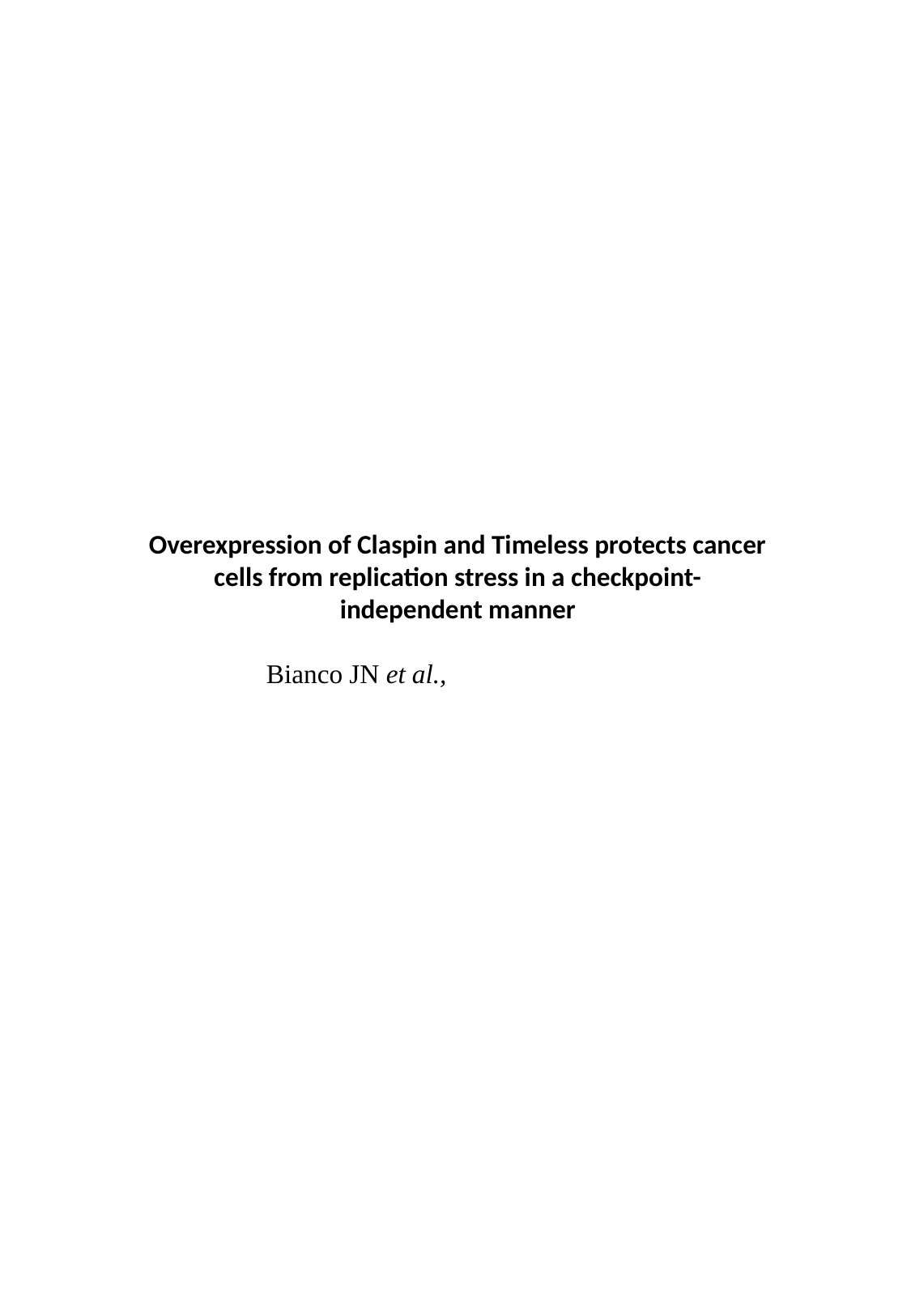

Overexpression of Claspin and Timeless protects cancer cells from replication stress in a checkpoint-independent manner
	Bianco JN et al.,

## Slide 2
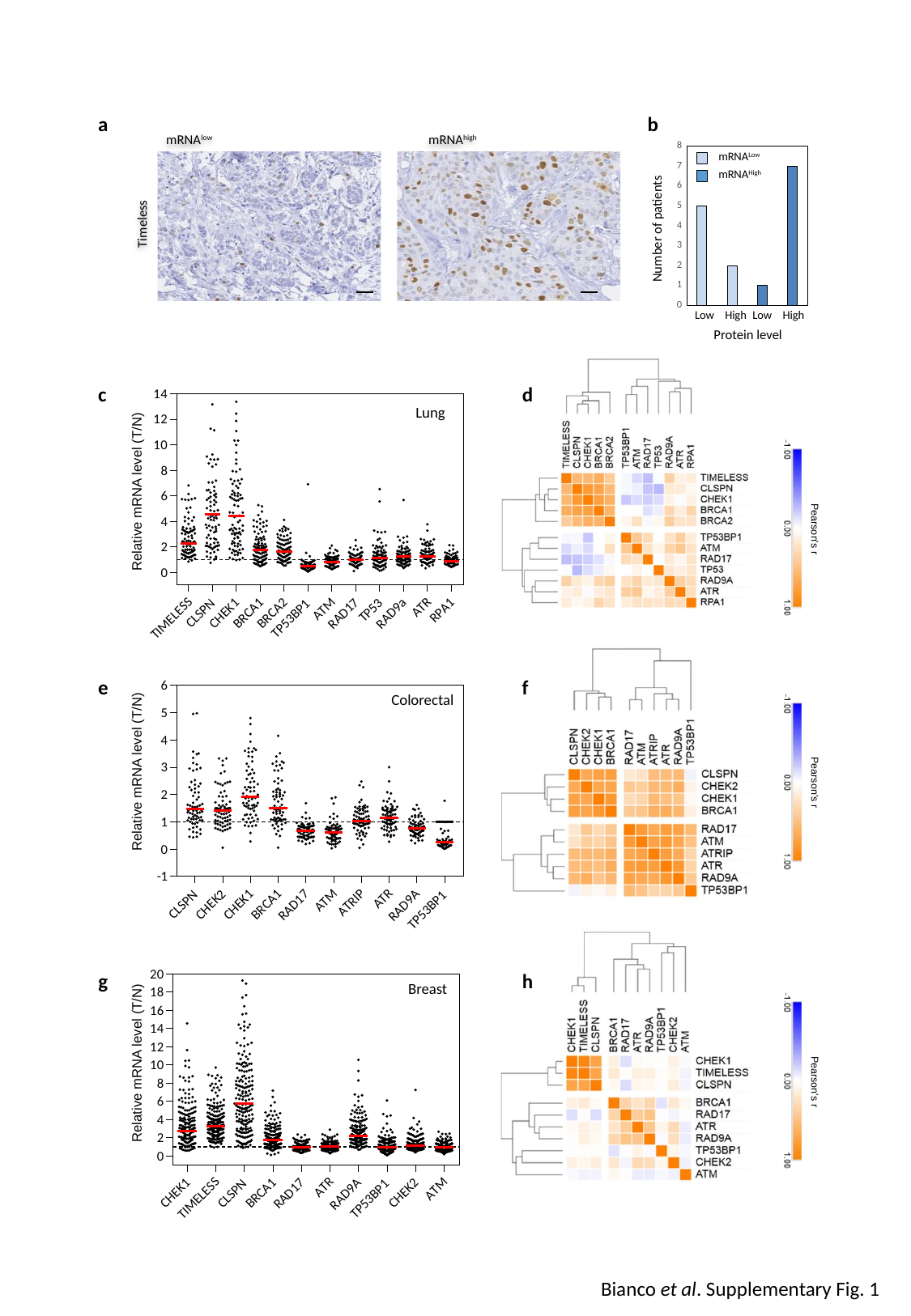

### Chart
| Category | |
|---|---|a
b
mRNAlow
mRNAhigh
mRNALow
mRNAHigh
Timeless
Number of patients
Low
High
Low
High
Protein level
c
d
Lung
Relative mRNA level (T/N)
Pearson's r
e
f
Colorectal
Pearson's r
Relative mRNA level (T/N)
g
h
Breast
Relative mRNA level (T/N)
Pearson's r
Bianco et al. Supplementary Fig. 1

## Slide 3
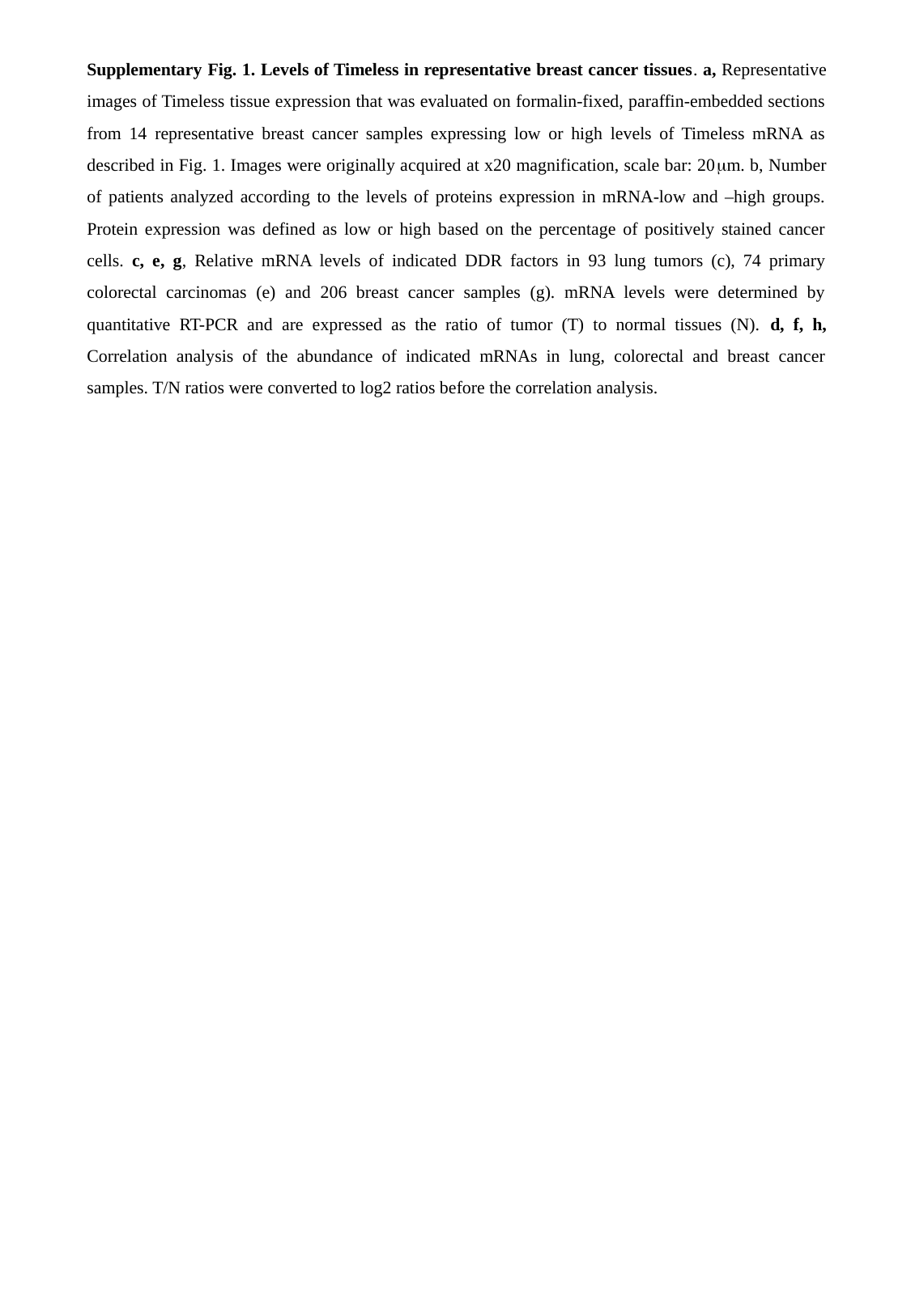

Supplementary Fig. 1. Levels of Timeless in representative breast cancer tissues. a, Representative images of Timeless tissue expression that was evaluated on formalin-fixed, paraffin-embedded sections from 14 representative breast cancer samples expressing low or high levels of Timeless mRNA as described in Fig. 1. Images were originally acquired at x20 magnification, scale bar: 20mm. b, Number of patients analyzed according to the levels of proteins expression in mRNA-low and –high groups. Protein expression was defined as low or high based on the percentage of positively stained cancer cells. c, e, g, Relative mRNA levels of indicated DDR factors in 93 lung tumors (c), 74 primary colorectal carcinomas (e) and 206 breast cancer samples (g). mRNA levels were determined by quantitative RT-PCR and are expressed as the ratio of tumor (T) to normal tissues (N). d, f, h, Correlation analysis of the abundance of indicated mRNAs in lung, colorectal and breast cancer samples. T/N ratios were converted to log2 ratios before the correlation analysis.

## Slide 4
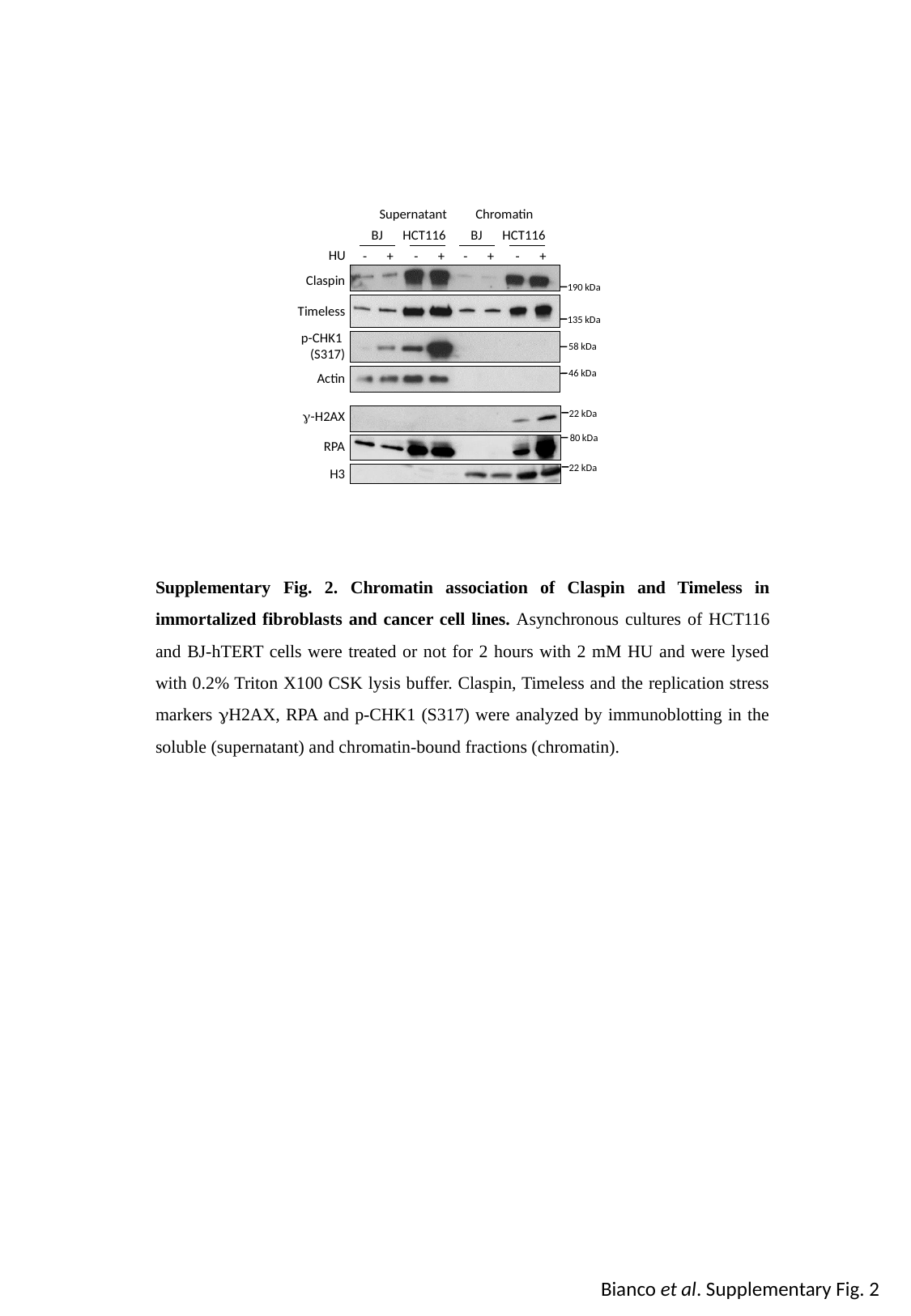

Supernatant
Chromatin
BJ
HCT116
BJ
HCT116
HU
-
+
-
+
-
+
-
+
Claspin
Timeless
p-CHK1
(S317)
Actin
g-H2AX
RPA
H3
190 kDa
135 kDa
58 kDa
46 kDa
22 kDa
80 kDa
22 kDa
Supplementary Fig. 2. Chromatin association of Claspin and Timeless in immortalized fibroblasts and cancer cell lines. Asynchronous cultures of HCT116 and BJ-hTERT cells were treated or not for 2 hours with 2 mM HU and were lysed with 0.2% Triton X100 CSK lysis buffer. Claspin, Timeless and the replication stress markers gH2AX, RPA and p-CHK1 (S317) were analyzed by immunoblotting in the soluble (supernatant) and chromatin-bound fractions (chromatin).
Bianco et al. Supplementary Fig. 2

## Slide 5
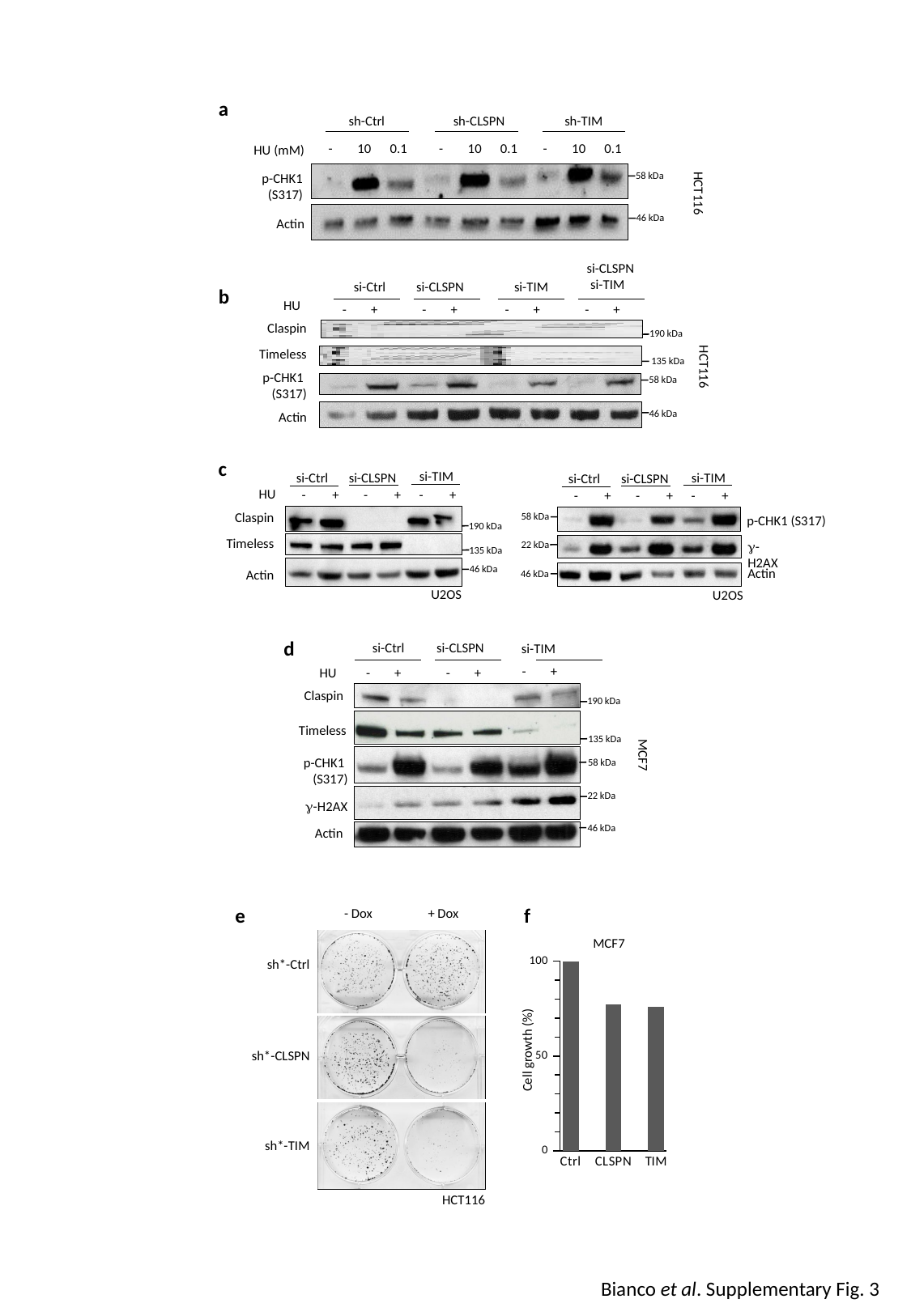

a
sh-Ctrl
sh-CLSPN
sh-TIM
-
10
0.1
-
10
0.1
-
10
0.1
 HU (mM)
58 kDa
p-CHK1
(S317)
HCT116
46 kDa
Actin
 si-CLSPN
si-TIM
 si-Ctrl
si-CLSPN
 si-TIM
b
HU
 - +
 - +
 - +
 - +
Claspin
190 kDa
Timeless
135 kDa
HCT116
p-CHK1
(S317)
58 kDa
46 kDa
Actin
c
si-TIM
si-TIM
si-CLSPN
si-Ctrl
si-CLSPN
si-Ctrl
HU
-
+
-
+
-
+
-
+
-
+
-
+
Claspin
58 kDa
p-CHK1 (S317)
190 kDa
Timeless
g-H2AX
22 kDa
135 kDa
46 kDa
Actin
Actin
46 kDa
U2OS
U2OS
d
 si-Ctrl
si-CLSPN
 si-TIM
 - +
HU
 - +
 - +
Claspin
190 kDa
Timeless
135 kDa
MCF7
p-CHK1
(S317)
58 kDa
22 kDa
g-H2AX
46 kDa
Actin
e
f
- Dox
+ Dox
sh*-Ctrl
sh*-CLSPN
sh*-TIM
MCF7
### Chart
| Category | |
|---|---|
| Ctrl | 100.0 |
| | None |
| CLSPN | 77.38079627384106 |
| | None |
| TIM | 75.89350255409403 |Cell growth (%)
HCT116
Bianco et al. Supplementary Fig. 3

## Slide 6
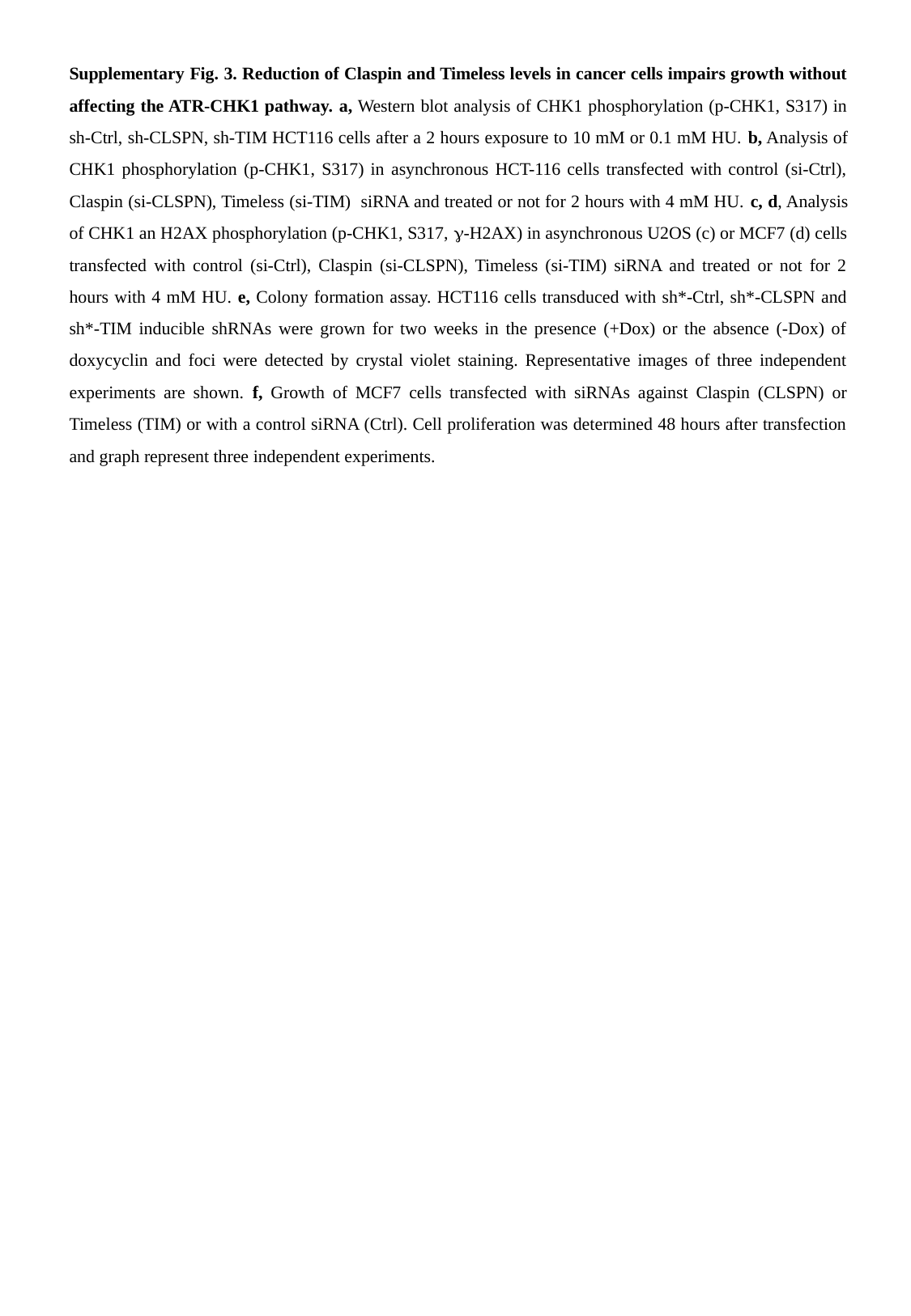

Supplementary Fig. 3. Reduction of Claspin and Timeless levels in cancer cells impairs growth without affecting the ATR-CHK1 pathway. a, Western blot analysis of CHK1 phosphorylation (p-CHK1, S317) in sh-Ctrl, sh-CLSPN, sh-TIM HCT116 cells after a 2 hours exposure to 10 mM or 0.1 mM HU. b, Analysis of CHK1 phosphorylation (p-CHK1, S317) in asynchronous HCT-116 cells transfected with control (si-Ctrl), Claspin (si-CLSPN), Timeless (si-TIM) siRNA and treated or not for 2 hours with 4 mM HU. c, d, Analysis of CHK1 an H2AX phosphorylation (p-CHK1, S317, g-H2AX) in asynchronous U2OS (c) or MCF7 (d) cells transfected with control (si-Ctrl), Claspin (si-CLSPN), Timeless (si-TIM) siRNA and treated or not for 2 hours with 4 mM HU. e, Colony formation assay. HCT116 cells transduced with sh*-Ctrl, sh*-CLSPN and sh*-TIM inducible shRNAs were grown for two weeks in the presence (+Dox) or the absence (-Dox) of doxycyclin and foci were detected by crystal violet staining. Representative images of three independent experiments are shown. f, Growth of MCF7 cells transfected with siRNAs against Claspin (CLSPN) or Timeless (TIM) or with a control siRNA (Ctrl). Cell proliferation was determined 48 hours after transfection and graph represent three independent experiments.

## Slide 7
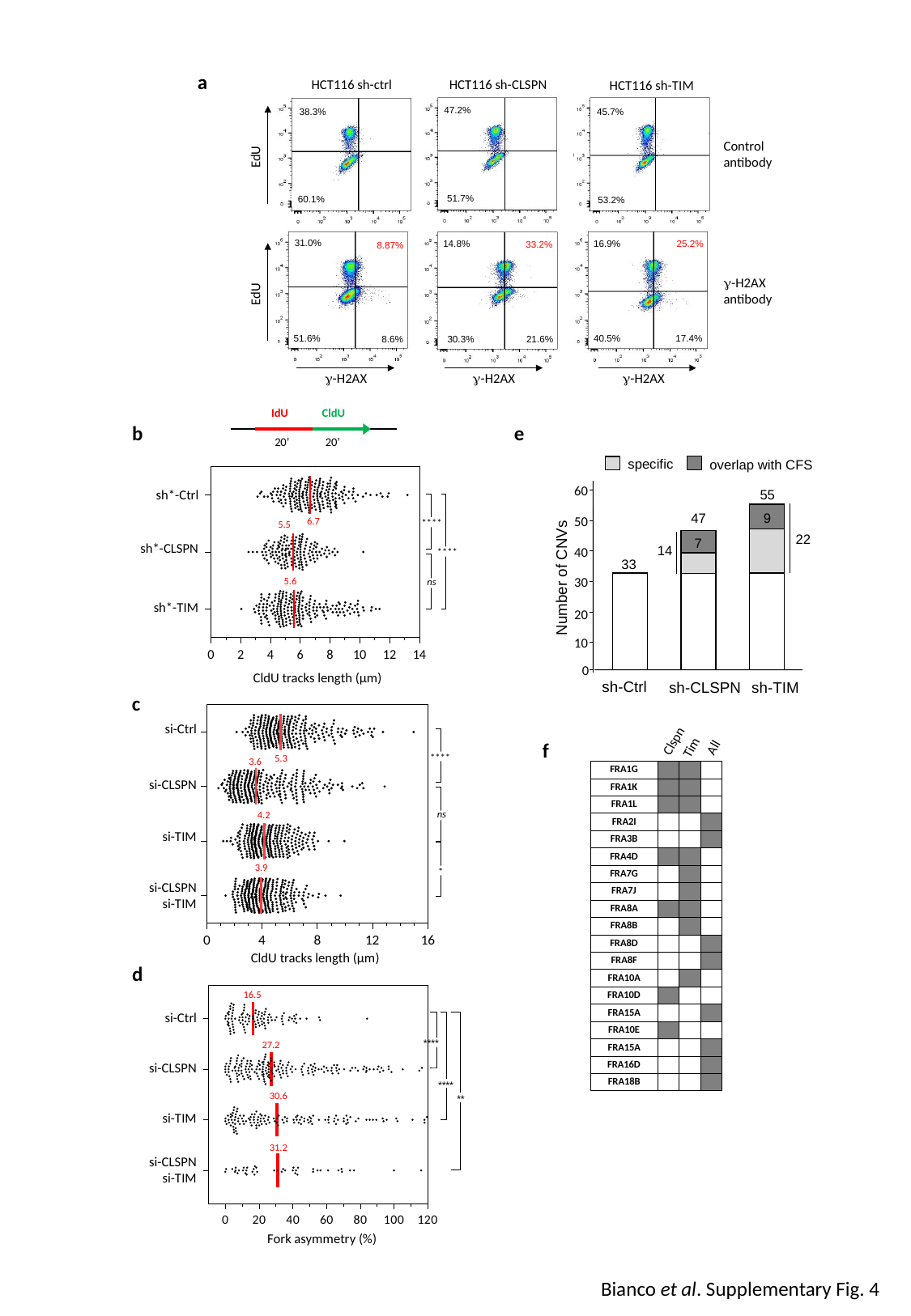

a
HCT116 sh-ctrl
HCT116 sh-CLSPN
HCT116 sh-TIM
47.2%
45.7%
38.3%
Control
antibody
EdU
51.7%
60.1%
53.2%
31.0%
25.2%
16.9%
14.8%
33.2%
8.87%
EdU +
gH2AX -
31%
EdU +
gH2AX +
8,87%
EdU +
gH2AX -
16,9%
EdU +
gH2AX +
25,2%
g-H2AX
antibody
EdU
51.6%
40.5%
17.4%
21.6%
8.6%
30.3%
g-H2AX
g-H2AX
g-H2AX
IdU
CldU
20’
20’
b
sh*-Ctrl
6.7
****
5.5
sh*-CLSPN
****
5.6
ns
sh-TIM
sh*-TIM
CldU tracks length (µm)
c
si-Ctrl
****
ns
*
5.3
3.6
si-CLSPN
4.2
si-TIM
3.9
si-CLSPN
si-TIM
CldU tracks length (µm)
d
16.5
si-Ctrl
****
27.2
si-CLSPN
****
30.6
**
si-TIM
31.2
si-CLSPN
si-TIM
Fork asymmetry (%)
e
specific
overlap with CFS
55
60
47
9
50
22
7
14
40
33
Number of CNVs
30
20
10
0
sh-Ctrl
sh-CLSPN
sh-TIM
Clspn
f
Tim
All
| FRA1G | 1 | 1 | |
| --- | --- | --- | --- |
| FRA1K | 1 | 1 | |
| FRA1L | 1 | 1 | |
| FRA2I | | | 1 |
| FRA3B | | | 1 |
| FRA4D | 1 | 1 | |
| FRA7G | | 1 | |
| FRA7J | | 1 | |
| FRA8A | 1 | 1 | |
| FRA8B | | 1 | |
| FRA8D | | | 1 |
| FRA8F | | | 1 |
| FRA10A | | 1 | |
| FRA10D | 1 | | |
| FRA15A | | | 1 |
| FRA10E | 1 | | |
| FRA15A | | | 1 |
| FRA16D | | | 1 |
| FRA18B | | | 1 |
Bianco et al. Supplementary Fig. 4

## Slide 8
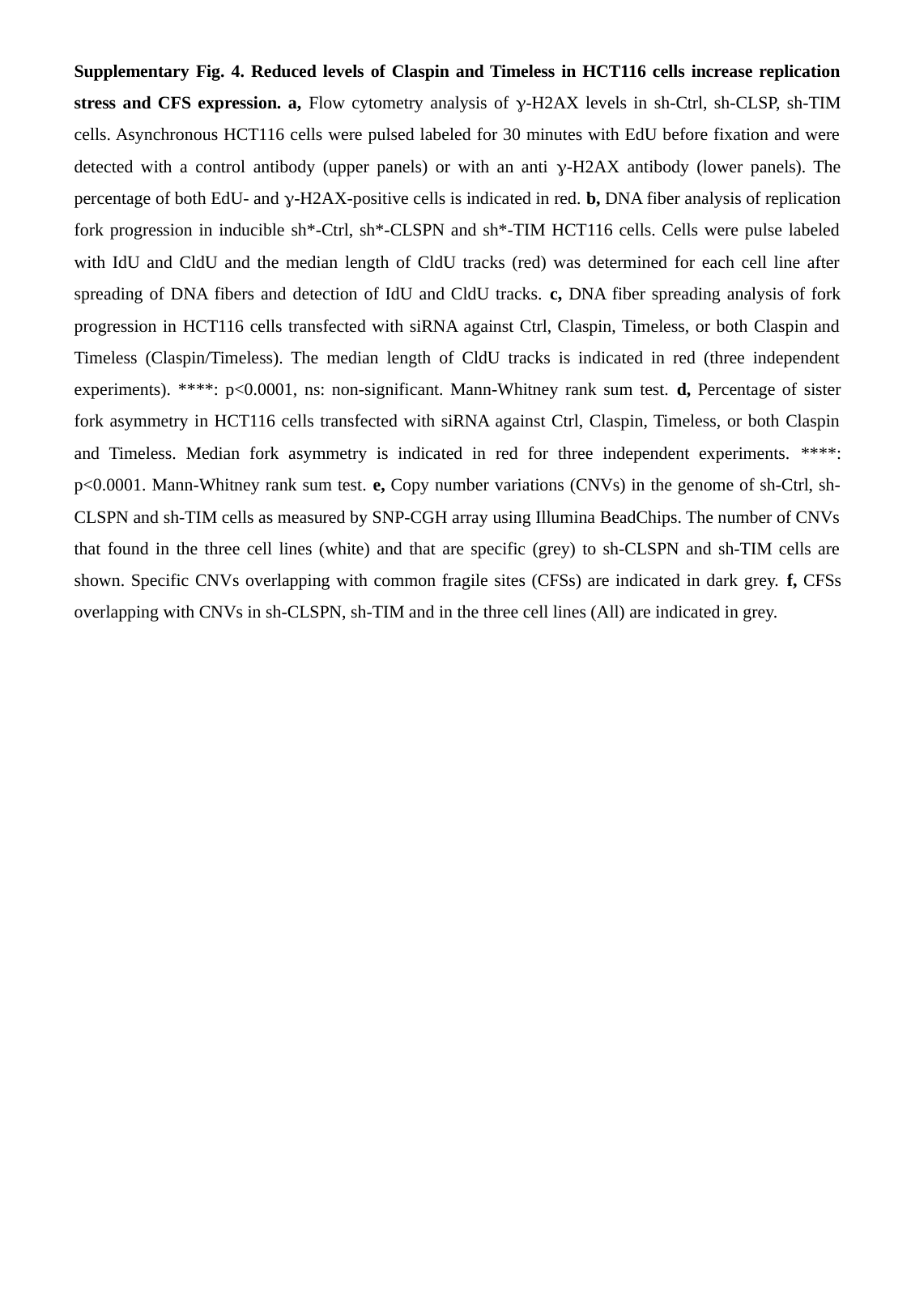

Supplementary Fig. 4. Reduced levels of Claspin and Timeless in HCT116 cells increase replication stress and CFS expression. a, Flow cytometry analysis of g-H2AX levels in sh-Ctrl, sh-CLSP, sh-TIM cells. Asynchronous HCT116 cells were pulsed labeled for 30 minutes with EdU before fixation and were detected with a control antibody (upper panels) or with an anti g-H2AX antibody (lower panels). The percentage of both EdU- and g-H2AX-positive cells is indicated in red. b, DNA fiber analysis of replication fork progression in inducible sh*-Ctrl, sh*-CLSPN and sh*-TIM HCT116 cells. Cells were pulse labeled with IdU and CldU and the median length of CldU tracks (red) was determined for each cell line after spreading of DNA fibers and detection of IdU and CldU tracks. c, DNA fiber spreading analysis of fork progression in HCT116 cells transfected with siRNA against Ctrl, Claspin, Timeless, or both Claspin and Timeless (Claspin/Timeless). The median length of CldU tracks is indicated in red (three independent experiments). ****: p<0.0001, ns: non-significant. Mann-Whitney rank sum test. d, Percentage of sister fork asymmetry in HCT116 cells transfected with siRNA against Ctrl, Claspin, Timeless, or both Claspin and Timeless. Median fork asymmetry is indicated in red for three independent experiments. ****: p<0.0001. Mann-Whitney rank sum test. e, Copy number variations (CNVs) in the genome of sh-Ctrl, sh-CLSPN and sh-TIM cells as measured by SNP-CGH array using Illumina BeadChips. The number of CNVs that found in the three cell lines (white) and that are specific (grey) to sh-CLSPN and sh-TIM cells are shown. Specific CNVs overlapping with common fragile sites (CFSs) are indicated in dark grey. f, CFSs overlapping with CNVs in sh-CLSPN, sh-TIM and in the three cell lines (All) are indicated in grey.

## Slide 9
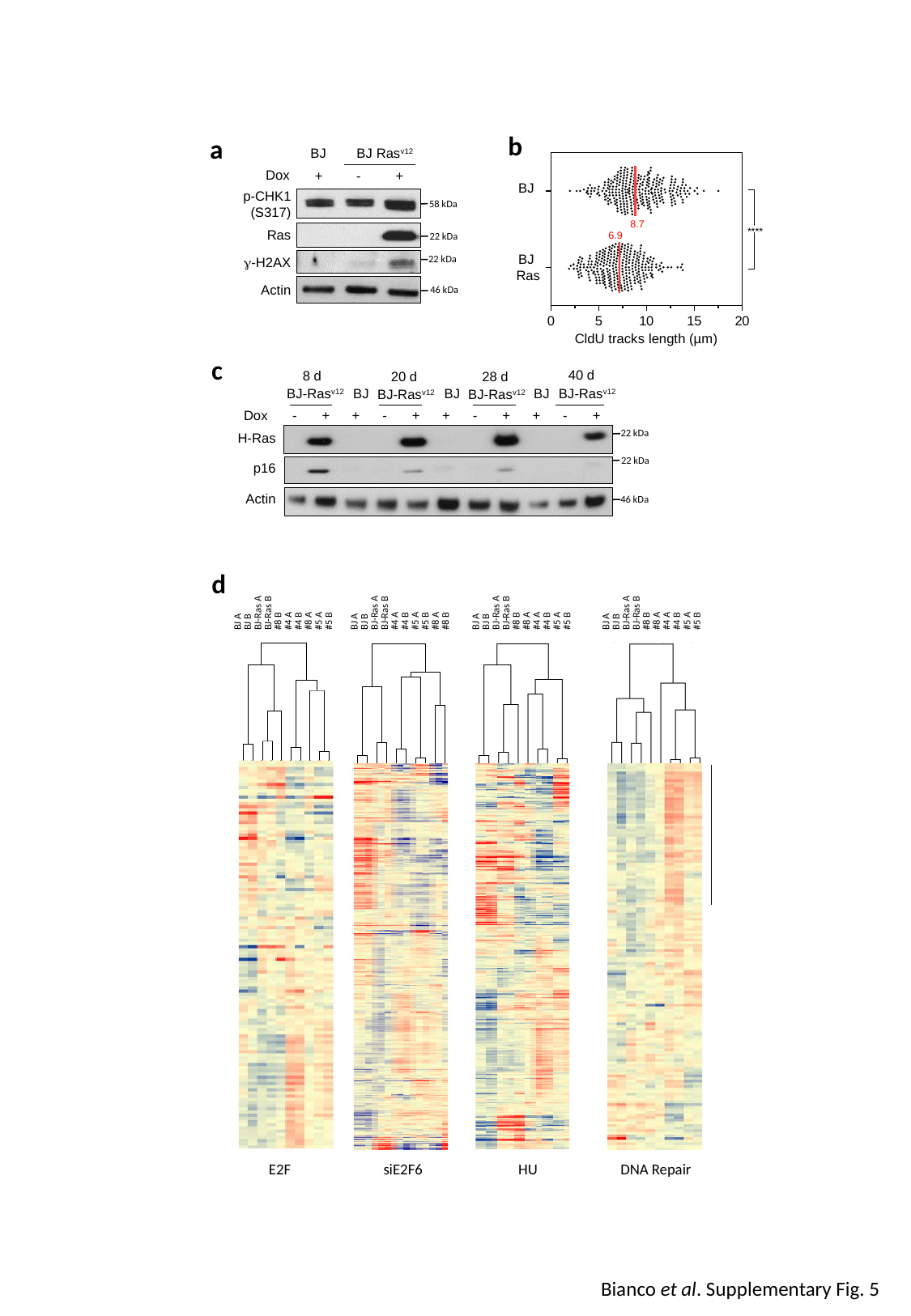

b
BJ
8.7
****
6.9
BJ
Ras
CldU tracks length (µm)
a
BJ
BJ Rasv12
Dox
+
-
+
p-CHK1
(S317)
58 kDa
Ras
22 kDa
22 kDa
g-H2AX
Actin
46 kDa
c
40 d
8 d
20 d
28 d
BJ-Rasv12
BJ
BJ
BJ
BJ-Rasv12
BJ-Rasv12
BJ-Rasv12
Dox
-
+
+
-
+
+
-
+
+
-
+
22 kDa
H-Ras
22 kDa
p16
Actin
46 kDa
BJ A
BJ B
BJ-Ras A
BJ-Ras B
#4 A
#4 B
#5 A
#5 B
#8 A
#8 B
BJ A
BJ B
BJ-Ras A
BJ-Ras B
#8 B
#8 A
#4 A
#4 B
#5 A
#5 B
BJ A
BJ B
BJ-Ras A
BJ-Ras B
#8 B
#8 A
#4 A
#4 B
#5 A
#5 B
BJ A
BJ B
BJ-Ras A
BJ-Ras B
#8 B
#4 A
#4 B
#8 A
#5 A
#5 B
d
E2F
siE2F6
HU
DNA Repair
Bianco et al. Supplementary Fig. 5

## Slide 10
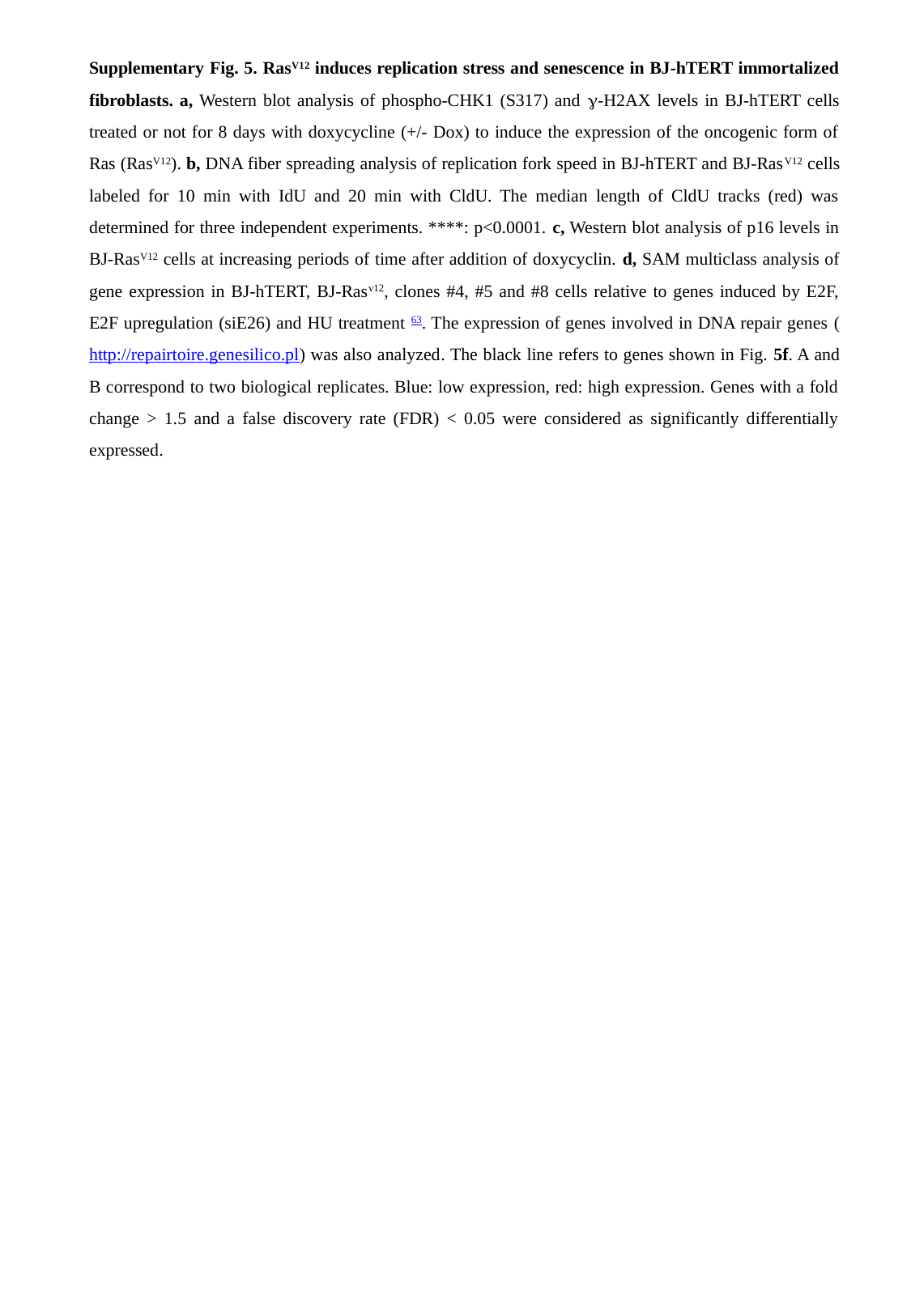

Supplementary Fig. 5. RasV12 induces replication stress and senescence in BJ-hTERT immortalized fibroblasts. a, Western blot analysis of phospho-CHK1 (S317) and g-H2AX levels in BJ-hTERT cells treated or not for 8 days with doxycycline (+/- Dox) to induce the expression of the oncogenic form of Ras (RasV12). b, DNA fiber spreading analysis of replication fork speed in BJ-hTERT and BJ-RasV12 cells labeled for 10 min with IdU and 20 min with CldU. The median length of CldU tracks (red) was determined for three independent experiments. ****: p<0.0001. c, Western blot analysis of p16 levels in BJ-RasV12 cells at increasing periods of time after addition of doxycyclin. d, SAM multiclass analysis of gene expression in BJ-hTERT, BJ-Rasv12, clones #4, #5 and #8 cells relative to genes induced by E2F, E2F upregulation (siE26) and HU treatment 63. The expression of genes involved in DNA repair genes (http://repairtoire.genesilico.pl) was also analyzed. The black line refers to genes shown in Fig. 5f. A and B correspond to two biological replicates. Blue: low expression, red: high expression. Genes with a fold change > 1.5 and a false discovery rate (FDR) < 0.05 were considered as significantly differentially expressed.

## Slide 11
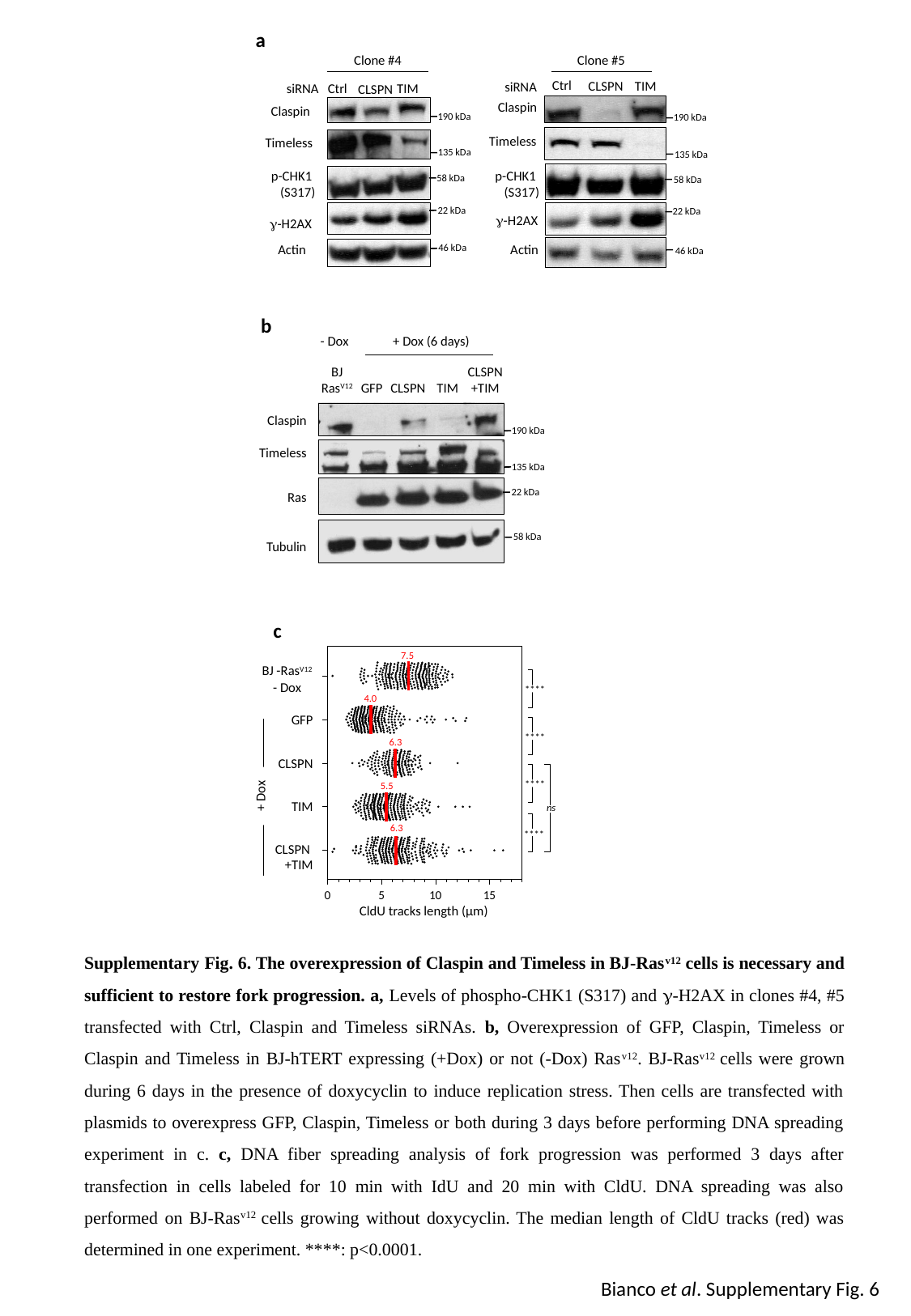

a
Clone #4
Clone #5
Ctrl
CLSPN
TIM
siRNA
Claspin
Timeless
p-CHK1
(S317)
g-H2AX
Actin
190 kDa
135 kDa
58 kDa
22 kDa
46 kDa
Ctrl
TIM
siRNA
CLSPN
Claspin
190 kDa
Timeless
135 kDa
p-CHK1
(S317)
58 kDa
22 kDa
g-H2AX
Actin
46 kDa
b
- Dox
+ Dox (6 days)
BJ RasV12
CLSPN
+TIM
GFP
CLSPN
TIM
Claspin
190 kDa
Timeless
135 kDa
22 kDa
Ras
58 kDa
Tubulin
c
7.5
BJ -RasV12
- Dox
****
4.0
GFP
****
6.3
CLSPN
****
5.5
+ Dox
TIM
ns
6.3
****
CLSPN
+TIM
CldU tracks length (µm)
Supplementary Fig. 6. The overexpression of Claspin and Timeless in BJ-Rasv12 cells is necessary and sufficient to restore fork progression. a, Levels of phospho-CHK1 (S317) and g-H2AX in clones #4, #5 transfected with Ctrl, Claspin and Timeless siRNAs. b, Overexpression of GFP, Claspin, Timeless or Claspin and Timeless in BJ-hTERT expressing (+Dox) or not (-Dox) Rasv12. BJ-Rasv12 cells were grown during 6 days in the presence of doxycyclin to induce replication stress. Then cells are transfected with plasmids to overexpress GFP, Claspin, Timeless or both during 3 days before performing DNA spreading experiment in c. c, DNA fiber spreading analysis of fork progression was performed 3 days after transfection in cells labeled for 10 min with IdU and 20 min with CldU. DNA spreading was also performed on BJ-Rasv12 cells growing without doxycyclin. The median length of CldU tracks (red) was determined in one experiment. ****: p<0.0001.
Bianco et al. Supplementary Fig. 6
