# Supplementary material for: Overexpression of Claspin and Timeless protects cancer cells from replication stress in a checkpoint-independent manner
Source: Nat Commun. 2019 Feb 22;10:910. doi: 10.1038/s41467-019-08886-8 (PMC6385232; doi:10.1038/s41467-019-08886-8)
Supplement: Supplementary file 3 — Reporting Summary [file 41467_2019_8886_MOESM3_ESM.pdf]

nature research

Corresponding author(s): TOURRIERE Hélène, PASERO Philippe

Reporting Summary

Nature Research wishes to improve the reproducibility of the work that we publish. This form provides structure for consistency and transparency in reporting. For further information on Nature Research policies, see Authors & Referees and the Editorial Policy Checklist.

Statistical parameters

When statistical analyses are reported, confirm that the following items are present in the relevant location (e.g. figure legend, table legend, main text, or Methods section).

n/a	Confirmed	
☐	☒	The exact sample size (*n*) for each experimental group/condition, given as a discrete number and unit of measurement
☐	☒	An indication of whether measurements were taken from distinct samples or whether the same sample was measured repeatedly
☐	☒	The statistical test(s) used AND whether they are one- or two-sided<br>*Only common tests should be described solely by name; describe more complex techniques in the Methods section.*
☐	☒	A description of all covariates tested
☐	☒	A description of any assumptions or corrections, such as tests of normality and adjustment for multiple comparisons
☐	☒	A full description of the statistics including central tendency (e.g. means) or other basic estimates (e.g. regression coefficient) AND variation (e.g. standard deviation) or associated estimates of uncertainty (e.g. confidence intervals)
☐	☒	For null hypothesis testing, the test statistic (e.g. *F*, *t*, *r*) with confidence intervals, effect sizes, degrees of freedom and *P* value noted<br>*Give P values as exact values whenever suitable.*
☐	☒	For Bayesian analysis, information on the choice of priors and Markov chain Monte Carlo settings
☐	☒	For hierarchical and complex designs, identification of the appropriate level for tests and full reporting of outcomes
☐	☒	Estimates of effect sizes (e.g. Cohen's *d*, Pearson's *r*), indicating how they were calculated
☐	☒	Clearly defined error bars<br>*State explicitly what error bars represent (e.g. SD, SE, CI)*

Our web collection on statistics for biologists may be useful.

Software and code

Policy information about availability of computer code

Data collection	*Provide a description of all commercial, open source and custom code used to collect the data in this study, specifying the version used OR state that no software was used.*
Data analysis	*Provide a description of all commercial, open source and custom code used to analyse the data in this study, specifying the version used OR state that no software was used.*

For manuscripts utilizing custom algorithms or software that are central to the research but not yet described in published literature, software must be made available to editors/reviewers upon request. We strongly encourage code deposition in a community repository (e.g. GitHub). See the Nature Research guidelines for submitting code & software for further information.

Data

Policy information about availability of data

All manuscripts must include a data availability statement. This statement should provide the following information, where applicable:

- Accession codes, unique identifiers, or web links for publicly available datasets
- A list of figures that have associated raw data
- A description of any restrictions on data availability

The datasets generated during and/or analysed during the current study are available in the [NAME] repository, [PERSISTENT WEB LINK TO DATASETS]• The

datasets generated during and/or analysed during the current study are available from the corresponding author on reasonable request. Row data associated to figures 2 to 6 are presented in supplementary datas files.

Field-specific reporting

Please select the best fit for your research. If you are not sure, read the appropriate sections before making your selection.

☒ Life sciences ☐ Behavioural & social sciences ☐ Ecological, evolutionary & environmental sciences

For a reference copy of the document with all sections, see nature.com/authors/policies/ReportingSummary-flat.pdf

Life sciences study design

All studies must disclose on these points even when the disclosure is negative.

Sample size	The sample sizes of different experiments, including DNA fiber spreanding and immunofluorescence microscopy, were chosen according to the previous studies published by other laboratories.
Data exclusions	No data exclusion
Replication	All experiments were reliably reproduced.
Randomization	The samples (cell lines) were allocated into control and various experimental groups according to the expression of wild-type or claspin/ timeless depleted samples.
Blinding	Blinded experiments were performed for DNA fiber analyses.

Reporting for specific materials, systems and methods

Materials & experimental systems

n/a	Involved in the study	
☐	☒	Unique biological materials
☐	☒	Antibodies
☐	☒	Eukaryotic cell lines
☒	☐	Palaeontology
☒	☐	Animals and other organisms
☒	☐	Human research participants

Methods

n/a	Involved in the study	
☒	☐	ChIP-seq
☐	☒	Flow cytometry
☒	☐	MRI-based neuroimaging

Unique biological materials

Policy information about availability of materials

Obtaining unique materials	All unique materials used are readily available from the authors or from standard commercial sources

Antibodies

Antibodies used

The source and the dilution for all antibodies used in this study are included in the manuscript in Methods.
Antibodies used in human cells-
Antibody Catalog No. Dilution
Mouse anti-BrdU clone B44 347580, BD Biosciences 1/100
Rat anti-BrdU clone BU1/75 ABC117-7513, Eurobio Abcys 1/100
anti-BrdU for ssDNA foci 347580, BD Biosciences 1/20
anti-ssDNA MAB3868, Millipore 1/250
anti-pCHK1 (S345) 2348, Cell signaling 1/1000
Phospho-Chk1 (Ser317) Cell Signaling/ozyme, 2344S 1/1000
anti-g-H2AX (S139) 05-636, Millipore 1/1000
anti-actin A4700, Sigma 1/500
anti-RPA1 Ab79398, abcam 1/300
anti-claspin (Halazonetis lab gift) 1/50
anti-Timeless interchim, CK-1290 1/2000

anti-ATR abcam, ab-10312 1/2000
anti-Rad17 MBL, K0120-03 1/1000
anti-Rad9 santa cruz biotechnology, sc-8324 1/1000
anti-chk1 Cell Signaling/ozyme, 2360 1/1000
anti-cdc25A santa cruz biotechnology,sc-7389 1/500
anti-ras BD transduction lab, 610002 1/500
Goat anti-rat Alexa 488 Molecular Probes, A11006 1/100
Goat anti-mouse IgG1Alexa 546 Molecular Probes, A21123 1/100
Goat anti-Mouse IgG2a Alexa 647 Molecular Probes, A21241 1/50

Validation	*Describe the validation of each primary antibody for the species and application, noting any validation statements on the manufacturer's website, relevant citations, antibody profiles in online databases, or data provided in the manuscript.*

Eukaryotic cell lines

Policy information about cell lines

Cell line source(s)	The human HCT116 colorectal carcinoma cell line was provided by A. Coquelle (IRCM, Montpellier, France). Normal human fibroblasts IMR90 and BJ-hTERT were a gift of J-M Lemaitre (IGF, Montpellier, France). MCF7 breast cancer cells were provided M. Piechaczyk (IGMM, Montpellier, France). HeLa cervical cancer cells and U2OS cells were a gift of M. Benkirane (IGH, France),
Authentication	None of the cell line used have been authenticated
Mycoplasma contamination	All the cell lines are mycoplasma-free. They have been tested for mycoplasma contamination regularly using MycoAlert Mycoplasma Detection Kit (LONZA).
Commonly misidentified lines (See ICLAC register)	No commonly misidentified cell lines were used in the study.

Flow Cytometry

Plots

Confirm that:

☒ The axis labels state the marker and fluorochrome used (e.g. CD4-FITC).

☒ The axis scales are clearly visible. Include numbers along axes only for bottom left plot of group (a 'group' is an analysis of identical markers).

☒ All plots are contour plots with outliers or pseudocolor plots.

☒ A numerical value for number of cells or percentage (with statistics) is provided.

Methodology

Sample preparation	Cells were fixed in ethanol and stainned with DAPI prior to cell sorting.<br>For S phase progression, cells were labelled with EdU, fixed and permeabilized. After Click-it reaction, cells were proceeded to DAPI stainning and RNase A treament before FACS analysis.
Instrument	For cell sorting, FACSAria<br>For S phase progression, Miltenyi MACSQuant
Software	FlowJo
Cell population abundance	For cell sorting, 20 millions cells were collected.<br>For S phase progression, 100, 000 cells were analyzed.
Gating strategy	Cells were gated first with SSC and FSC. then single cell population was further gated for cell cycle analyses.

☐ Tick this box to confirm that a figure exemplifying the gating strategy is provided in the Supplementary Information.
